# Impact and Cost-Effectiveness of Culture for Diagnosis of Tuberculosis in HIV-Infected Brazilian Adults

**DOI:** 10.1371/journal.pone.0004057

**Published:** 2008-12-29

**Authors:** David W. Dowdy, Maria C. Lourenço, Solange C. Cavalcante, Valeria Saraceni, Bonnie King, Jonathan E. Golub, David Bishai, Betina Durovni, Richard E. Chaisson, Susan E. Dorman

**Affiliations:** 1 Center for Tuberculosis Research, Johns Hopkins University School of Medicine, Baltimore, Maryland, United States of America; 2 Department of Epidemiology, Johns Hopkins Bloomberg School of Public Health, Baltimore, Maryland, United States of America; 3 Mycobacteriology Laboratory, Fundação Oswaldo Cruz, Rio de Janeiro, Brazil; 4 Communicable Diseases Program, Municipal Health Secretariat, Rio de Janeiro, Brazil; 5 Department of Population and Family Health Sciences, Johns Hopkins Bloomberg School of Public Health, Baltimore, Maryland, United States of America; 6 Department of International Health, Johns Hopkins Bloomberg School of Public Health, Baltimore, Maryland, United States of America; McGill University, Canada

## Abstract

**Background:**

Culture of *Mycobacterium tuberculosis* currently represents the closest “gold standard” for diagnosis of tuberculosis (TB), but operational data are scant on the impact and cost-effectiveness of TB culture for human immunodeficiency (HIV-) infected individuals in resource-limited settings.

**Methodology/Principal Findings:**

We recorded costs, laboratory results, and dates of initiating TB therapy in a centralized TB culture program for HIV-infected patients in Rio de Janeiro, Brazil, constructing a decision-analysis model to estimate the incremental cost-effectiveness of TB culture from the perspective of a public-sector TB control program. Of 217 TB suspects presenting between January 2006 and March 2008, 33 (15%) had culture-confirmed active tuberculosis; 23 (70%) were smear-negative. Among smear-negative, culture-positive patients, 6 (26%) began TB therapy before culture results were available, 11 (48%) began TB therapy after culture result availability, and 6 (26%) did not begin TB therapy within 180 days of presentation. The cost per negative culture was US$17.52 (solid media)–$23.50 (liquid media). Per 1,000 TB suspects and compared with smear alone, TB culture with solid media would avert an estimated eight TB deaths (95% simulation interval [SI]: 4, 15) and 37 disability-adjusted life years (DALYs) (95% SI: 13, 76), at a cost of $36 (95% SI: $25, $50) per TB suspect or $962 (95% SI: $469, $2642) per DALY averted. Replacing solid media with automated liquid culture would avert one further death (95% SI: −1, 4) and eight DALYs (95% SI: −4, 23) at $2751 per DALY (95% SI: $680, dominated). The cost-effectiveness of TB culture was more sensitive to characteristics of the existing TB diagnostic system than to the accuracy or cost of TB culture.

**Conclusions/Significance:**

TB culture is potentially effective and cost-effective for HIV-positive patients in resource-constrained settings. Reliable transmission of culture results to patients and integration with existing systems are essential.

## Introduction

Human immunodeficiency virus (HIV) infection dramatically increases the incidence, severity, and mortality risk of active tuberculosis (TB) [1]. Unfortunately, HIV also complicates TB diagnosis. In HIV-infected patients, sputum smear microscopy has an estimated sensitivity of 35% for active TB [2], and smear-negative TB is associated with worse clinical outcomes than smear-positive disease [3], [4]. Improved diagnosis of TB in HIV-infected individuals is recognized as an increasingly urgent priority [5].

While many promising novel TB diagnostics are being developed [6], [7], expanded use of TB culture may have an immediate impact on TB rates in high-burden countries [8], [9]. Culture of *Mycobacterium tuberculosis* from clinical specimens currently represents the closest “gold standard” for diagnosis of TB [5]. Despite routine use throughout the developed world, TB culture remains unavailable in most high-burden countries, largely due to expense and infrastructure requirements [10], [11]. However, a number of high-burden countries have now developed laboratory capacity to perform TB culture [12], and automated systems using liquid media are now available that may reduce the corresponding human resource requirement [13]. To date, operational data are scant on the impact and cost-effectiveness of programs performing TB culture for HIV-infected individuals in resource-limited settings [14], [15]. Thus, we evaluated costs, laboratory results, clinical events (e.g., initiation and completion of TB therapy), and projected clinical outcomes (e.g., TB mortality) at a centralized referral laboratory offering culture to HIV-positive TB suspects across 29 clinics in Rio de Janeiro, Brazil.

## Methods

### Objectives

Our objectives were to estimate the impact and cost-effectiveness of mycobacterial culture for the diagnosis of TB in an urban setting in Latin America. Our primary outcome was the incremental cost-effectiveness ratio (ICER), expressed as the cost, in 2006 US dollars, per disability-adjusted life year (DALY) averted.

### Study Design and Participants

We performed a field evaluation of centralized TB culture in the context of the TB/HIV in Rio (THRio) study, details of which have been published elsewhere [16]. Briefly, THRio is a cluster-randomized trial of tuberculin skin testing and isoniazid preventive therapy for HIV-positive patients, taking place at 29 municipal health clinics and hospitals in Rio de Janeiro, Brazil. In a “stepped-wedge” design, two clinics were randomized to begin receiving the study intervention on a bimonthly basis, until all clinics were phased-in over a period of 30 months. Thus, data on the first two clinics were available for the entire study period, and on the last two clinics only for the final month. Data on all enrolled patients, including dates of TB diagnoses and outcomes of TB treatment, were abstracted from medical charts and clinic records on a semi-annual basis.

As clinics were phased-in to receive the THRio study intervention, they also became eligible to order TB culture through a centralized mycobacteriology laboratory. At this time, physicians and nurses were given standardized requisition forms and briefly trained to order TB culture on all HIV-positive pulmonary TB suspects (i.e., excluding HIV-negative patients, asymptomatic patients, and patients already being treated for active TB), recommending collection of two specimens per patient. Specimens were then collected throughout the week and refrigerated (before processing) until delivery to the laboratory on a weekly basis by secure motorized transport. Results from the laboratory were reported back to clinics on a weekly basis; thus, positive culture results were reported before the results of species identification (which were reported on all culture-positive specimens). In addition, a study nurse coordinated lab-clinic communication (including immediate reporting of positive smear results in order to insure that smear results would be available if patients were told to return in one week) and performed ongoing training, data management, and quality assurance.

The present analysis includes all patients who met the following criteria: (a) presentation to a THRio intervention clinic with symptoms and/or signs compatible with pulmonary TB between January 1, 2006, and March 15, 2008; (b) confirmed HIV-positive; (c) not diagnosed with TB in the period between 15 and 365 days before presentation; (d) at least one acceptable specimen for TB culture submitted; and (e) data on culture result and clinical TB diagnosis available for 180 days beyond specimen submission. Laboratory data were analyzed and reported by diagnostic attempt, defined as a series of TB culture specimens submitted within any continuous 15-day period.

### Laboratory Procedures

All specimens were decontaminated using a commercialized N-acetyl-L-cysteine/NaOH preparation (Mycoprep™, BD Corporation, Franklin Lakes, NJ). Ziehl-Neelsen sputum smear microscopy was performed according to standard procedures [17]. Smears were prepared both before and after centrifugation [18], with a positive result taken as detection of 10 acid-fast bacilli (AFB) per 100 high-power fields on either examination. According to the standard practice of the Brazilian reference laboratory, TB culture on solid media was performed by inoculating 100 µL of decontaminated material into each of five Lowenstein-Jensen (L-J) slants: three with no added inhibitors, one with 500 mg/L p-nitrobenzoic acid [PNB], and one with 5 mg/L thiophen-2-carboxylic acid hydrazide [TCH]. Specimens were incubated at 37°C and examined on a weekly basis for eight weeks. All results were verified by Ziehl-Neelsen microscopy, and all slants showing growth of AFB (one per patient specimen) were sub-cultured and subjected to species identification with standard biochemical tests [17].

Culture in liquid media was performed using the Mycobacteria Growth Indicator Tube [MGIT™] 960 automated system (BD Corporation, Franklin Lakes, NJ) using 1 culture tube per specimen. Tubes marked as having growth by the automated system were manually assessed for AFB using Ziehl-Neelsen microscopy; all tubes positive for AFB were sub-cultured on L-J media and species identification was performed as above. For specimens showing growth of other organisms in liquid culture, centrifuged pellets from the original decontaminated sputa (which were frozen at −20°C) were subjected to repeat decontamination and re-inoculation into fresh tubes, from which the final culture result was then obtained.

A patient's culture result was considered contaminated only if all slants or tubes from all cultures from all specimens during a diagnostic attempt revealed growth of organisms other than AFB. Otherwise, a positive result was taken as a single colony on solid media, or a single positive tube using liquid media, that revealed AFB on Ziehl-Neelsen microscopy. The absence of colonies (L-J) or fluorescence (MGIT) on all non-contaminated specimens qualified as a negative result. All results were entered into a computerized laboratory database and linked with patient records from the THRio study.

### Cost-Effectiveness Analysis

In this study, an expanded TB culture program was initiated in a standing bacteriology laboratory with sufficient existing infrastructure (e.g., biosafety laboratory space, autoclaves), but without the resources (e.g., staff, equipment, supplies) for program initiation or maintenance. Reasoning that existing laboratory infrastructure may be a pre-condition for the cost-effective establishment of TB culture programs in many developing countries, we adopted the perspective of a public-sector TB control program with such infrastructure available, deciding whether to fund expanded TB culture. The reference scenario, therefore, is the situation in which the central laboratory does not perform TB culture despite capacity to do so; less than 5% of Brazilian laboratories that perform sputum smear microscopy currently perform TB culture [12]. In this reference scenario, we assume that all TB suspects have sputum smears performed at a local laboratory, at the same cost as if performed in the central laboratory.

Using decision analysis, we estimated the incremental cost-effectiveness of sputum smear microscopy plus expanded TB culture using (a) L-J solid media and (b) liquid media with MGIT, against the reference scenario of sputum smear microscopy alone. Secondary outcomes included incremental TB diagnoses made, incremental deaths averted, and incremental secondary infections averted. We performed our analysis using a hypothetical cohort of HIV-positive patients presenting to municipal health clinics with symptoms of pulmonary TB (e.g., cough of three weeks' duration).

Among patients with active TB, we assumed that TB culture offers no benefit among sputum-smear positive patients, and that a proportion of patients (as estimated by the World Health Organization, WHO [19]) are diagnosed with smear-negative TB on the basis of tools other than culture (e.g., chest X-ray, clinical judgment). Thus, TB culture offers benefit only to those patients who would otherwise go undiagnosed after utilization of all other available clinical tools. The cost of false-positive diagnosis was incorporated as the cost of TB treatment, plus a corresponding decrement in quality of life while on TB therapy. Patients with undiagnosed TB were assumed to experience a monthly mortality risk commensurate with the estimated case-fatality rate for untreated TB in HIV-positive Brazilian adults [19], assuming a constant proportion of deaths in each of 12 months. Such patients were also assumed to generate secondary TB infections at a defined monthly rate. Those secondary infections progressing rapidly to active TB were assumed to result in immediate loss of quality-adjusted life; latent infections and “tertiary” infections resulting from secondary cases were ignored. A Markov process was used to model the assumption that patients with undiagnosed TB would present to the same clinic for re-diagnosis, at a rate determined by the time interval between repeat specimen submissions, until diagnosis or death. The time horizon was taken as the life of the cohort, with future costs and DALYs discounted at 3% per year (i.e., costs and DALYs in future years are valued at 97% of their value in the preceding year).

Where possible, model parameter estimates were based on direct study data; otherwise, parameter estimates were obtained from the literature. Costs were obtained directly from laboratory budget records and staff interviews using an “ingredients” approach; only those incremental costs accruing to the municipal tuberculosis program for initiation and maintenance of TB culture were included. Certain items (e.g., freezers, incubators, laboratory chemicals) were not directly purchased by the municipal tuberculosis program; their costs were estimated according to Brazilian market values. Costs of capital items were annualized over their useful lives. Useful life was estimated according to WHO-CHOosing Interventions that are Cost-Effective (CHOICE) published values where available [20]; otherwise, laboratory equipment items (i.e., MGIT reader, centrifuge) were assumed to have a useful life of 10 years, while supplies (e.g., pipettes, reusable glassware) were assumed to last 5 years. Estimated test characteristics were based on actual field performance, using any positive culture for *M. tuberculosis* or initiation of TB treatment as the definition of a TB case. Outcomes and effectiveness were estimated by linkage of patient lab records to medical charts in the THRio database.

### Sensitivity and Uncertainty Analysis

We performed one-way sensitivity analyses on all model parameters, evaluating the impact on incremental cost-effectiveness (comparing TB culture with solid media to the baseline scenario) of a ±25% change in parameter value. To assess the impact of simultaneous changes in all model parameters, we also performed multivariate uncertanty analysis, varying all parameter estimates over beta distributions (for variables bounded between 0 and 1) or gamma distributions (for variables bounded between 0 and infinity), with means set to expected parameter values and standard deviations set to 12.5% of expected parameter values. The results of 10,000 simulations are presented as 95% simulation intervals, which correspond to the 2.5^th^ and 97.5^th^ percentile of simulated results.

### Ethics

This study operated under a waiver of informed consent, as it involved secondary collection of de-identified data after implementing the standard of care for TB diagnosis, as recommended by the City of Rio de Janeiro. This study was approved by the institutional review boards of the Johns Hopkins Medical Institutions and the City of Rio de Janeiro.

## Results

### Laboratory Outcomes and Treatment Initiation

**Figure 1:**
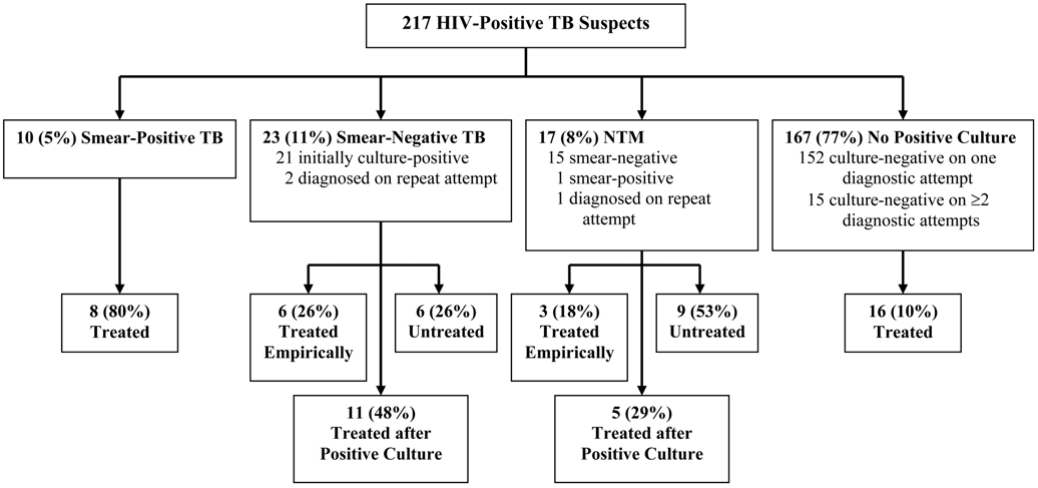
Patient Flow Diagram. In this diagram, “positive” denotes a positive result on any smear, slant, or tube, and “negative” denotes the absence of such a result, including if all specimens are contaminated. “Treated” denotes treatment for TB, and “treated empirically” refers to treatment initiated prior to the availability of culture results. HIV, human immunodeficiency virus; TB, tuberculosis; NTM, non-tuberculous mycobacteria.

A total of 217 eligible patients submitted 398 TB culture specimens during 235 diagnostic attempts, a mean of 1.69 specimens per attempt. Of these, 33 patients (15%) had *M. tuberculosis* isolated from at least one culture; 10 (30%) were smear-positive on initial evaluation. An additional 17 patients (7%) had cultures positive for non-tuberculous mycobacteria (NTM) (11 *M. fortuitum*, 4 *M. avium*, 1 *M. kansasii*, 1 *M. flavescens*), and 19 patients (9%) were treated for TB without a positive culture (Figure 1).

To minimize costs, we adopted different processing algorithms for solid versus liquid culture media. According to laboratory routine, five solid L-J slants were prepared from each specimen, and a new specimen was requested if all five slants showed contamination. By contrast, only a single MGIT tube was initially prepared for each specimen, but contaminated specimens were re-processed for repeat culture. Ignoring re-processing, sensitivity for culture-confirmed, smear-negative TB was identical (17/23 = 74%) for solid media (5 slants) versus MGIT (1 tube), but MGIT was faster (mean time to culture growth 20.0 versus 31.9 days, p = 0.0001) and more likely to detect non-tuberculous mycobacteria (NTM) (14/17 = 82% versus 8/17 = 47%, p = 0.03). Of MGIT specimens, 14% were contaminated, versus 9% for five L-J slants (p = 0.22). Re-processing MGIT specimens yielded two additional TB diagnoses; updated MGIT sensitivity was thus 83% (19/23). The sensitivity estimates used for the cost-effectiveness model are somewhat lower than these figures, since the cost-effectiveness model assumes that some smear-negative TB cases are also culture-negative.

Treatment for TB was initiated in 49 patients: 8/10 (80%) with smear-positive TB, 17/23 (74%) with culture-confirmed smear-negative TB, 8/17 (47%) with cultures positive for NTM, and 16/167 (10%) with negative smears and cultures (Figure 1). Among 17 patients with culture-confirmed smear-negative TB who were treated, six (35%) began treatment before culture results became available. Among eight treated patients with cultures positive for NTM, three (37.5%) began TB treatment before culture results were reported. Thus, 5/14 (36%) untreated NTM-positive patients were placed on TB therapy after receiving a positive culture result; these patients completed 81% (mean) of a full treatment course of a full treatment course before stopping therapy for TB. Although species identification results were reported promptly on all patients, 50% of patients with cultures positive for NTM completed a full course of TB therapy and were registered as TB cured or treatment completed, with no mention of an alternative diagnosis.”

### Cost and Cost-Effectiveness

We estimated the unit cost of TB culture under two different scenarios of laboratory throughput: observed throughput (eight patients/week) and estimated maximum throughput using existing equipment and staff (24 patients/week) (Table 1). The greatest single cost was the weekly transportation of specimens and results to each clinic. As throughput increased, culture tubes and reagents accounted for an increasing proportion of TB culture costs, particularly for MGIT. Table 2 presents other parameters used to model the cost-effectiveness of TB culture. Assuming eight patients per week, use of MGIT reduced staff time required for TB culture (i.e., excluding smear, senior oversight, etc.) by 8% compared to solid media (data not shown).

**Table 1:** Unit Cost Estimates for Expanded TB Culture Program (in 2006 US$).

Item/Category	8 Patients per Week		24 Patients per Week	
	Solid Media^a^	MGIT^b^	Solid Media^a^	MGIT^b^
*Variable Costs*				
Culture tubes and media	$0.59	$3.00	$0.59	$3.00
Decontamination reagents	$0.83	$0.83	$0.83	$0.83
Cryovials for pellet storage	$0.00	$0.81	$0.00	$0.81
Lab supplies (e.g., pipette tips, centrifuge tubes)	$0.53	$0.53	$0.53	$0.53
*Fixed Costs*				
Transportation^c^	$9.61	$8.57	$3.20	$2.86
Automated MGIT 960 reader	$0.00	$4.62	$0.00	$1.54
Laboratory personnel^c^	$4.21	$3.75	$1.40	$1.25
Lab supplies (e.g., mini-pipettes, vortex machine)^d^	$1.17	$1.00	$0.39	$0.33
Lab equipment (e.g., incubator, freezer)^d^	$0.59	$0.39	$0.20	$0.13
**Total cost per Negative Culture**	**$17.52**	**$23.50**	**$7.14**	**$11.28**
Confirmation/speciation	$7.90	$9.18	$7.82	$8.24
**Total cost per Positive Culture**	**$25.42**	**$32.68**	**$14.96**	**$19.52**

MGIT, Mycobacteria Growth Indicator Tube.

a Price per specimen, including five Lowenstein-Jensen slants (see Methods).

b Price per culture tube: one per specimen, re-inoculated if contaminated (see Methods).

c Differences between MGIT and solid media reflect higher volume of MGIT specimens (due to re-inoculation) at the same cost, thus reducing cost per MGIT specimen relative to solid media.

d Differences between MGIT and solid media partially reflect higher volume of MGIT specimens (due to re-inoculation) at the same cost.

**Table 2:** Parameter Estimates for Cost-Effectiveness Model.

Parameter	Value (Sensitivity Range)	Reference
*Study Characteristics*		
Number of clinics served	29 (22–36)	Study data
Number of TB suspects per week	8 (6–10)	Study data
Number of specimens per diagnostic attempt	1.69 (1.27–2)	Study data
Prevalence of TB among TB suspects^a^	0.19 (0.14–0.24)	Study data
*TB Diagnosis: Baseline*		
Sensitivity of sputum smear	0.30 (0.23–0.38)	Study data
Sensitivity of clinician diagnosis for smear-negative TB	0.56 (0.42–0.70)	[12]
Specificity of clinician diagnosis (including smear)	0.91 (0.68–1.0)	[29]
Days to repeat presentation if TB not diagnosed	113 (85–141)	Study data
*TB Diagnosis: Culture* b		
Contamination rate per diagnostic attempt		Study data
Solid media	0.09 (0.07–0.11)	
MGIT	0.008^c^ (0.006–0.011)	
Sensitivity per diagnostic attempt, smear-positive TB		Study data
Solid media	1.0 (0.75–1.0)	
MGIT	1.0 (0.75–1.0)	
Sensitivity per diagnostic attempt, smear-negative TB^a^		Study data
Solid media	0.64 (0.48–0.80)	
MGIT	0.68^c^ (0.51–0.85)	
Proportion of TB-negative cultures growing NTM (1−specificity for active TB)^a^		Study data
Solid media	0.06 (0.04–0.07)	
MGIT	0.10 (0.07–0.12)	
Days to first positive culture for smear-negative TB		Study data
Solid media	38 (29–48)	
MGIT	29 (22–36)	
Days from positive culture to TB treatment, mean	46 (34–58)	Study data
Proportion of NTM cultures triggering TB therapy	0.36 (0.27–0.45)	Study data
Proportion of TB-positive cultures triggering TB therapy	0.65 (0.49–0.81)	Study data
*Additional Cost Estimates*		
Cost of repeated diagnostic attempt (excluding culture)	$28 ($21–$35)	[30]
Cost of treating a single case of active TB	$516 ($387–$645)	[12]
Mean fraction of TB treatment course completed by patients with NTM infection who are treated for TB	0.81 (0.61–1.0)	Study data
*Outcomes and Transmission*		
Mortality rate of undiagnosed TB, per month	0.05 (0.04–0.06)	[19]
Life expectancy of patient diagnosed with TB, years	19.5 (14.6–24.4)	THRio data
Disability weight, HIV	0.24 (0.18–0.30)^d^	[31]
Disability weight, active TB	0.27 (0.20–0.34)	[31]
Quality of life decrement, treatment for TB	0.14 (0.10–0.17)^e^	Assumption
Secondary infections per smear-negative case, per year	2.9 (2.2–3.6)	[32], [33]
Proportion of secondary infections progressing rapidly to active TB	0.05 (0.04–0.06)	[32], [34]
TB mortality among secondary cases of active TB	0.13 (0.10–0.17)	[12]

TB, tuberculosis; MGIT, Mycobacteria Growth Indicator Tube; NTM, non-tuberculous.

mycobacteria; HIV, human immunodeficiency virus.

a The estimated number of true-positives includes all confirmed cases, plus a proportion of culture-negative cases, assuming a positive predictive value of 52% for clinician diagnosis based on the prevalence of TB and estimated sensitivity and specificity of clinical diagnosis.

b Solid media = five Lowenstein-Jensen slants per specimen; MGIT = one BBL® culture tube per specimen, re-inoculated if initially contaminated (see Methods). Sensitivity and specificity are calculated among diagnostic attempts in which at least one culture was not contaminated. Diagnostic attempts may include more than one culture specimen.

c The lower contamination rate and higher sensitivity for MGIT over solid media reflect the practice of re-processing contaminated MGIT specimens, whereas solid cultures were inoculated in parallel and not re-processed.

d Disability weight of 0.136 for HIV infection without AIDS [31], +0.1 for the burden of antiretroviral therapy.

e Assumes that TB treatment (lasting six months) results in 50% as much disability as active TB.

We estimated that, for every 1,000 HIV-positive TB suspects presenting to clinics without access to TB culture, 188 would have active TB, resulting in 73 secondary TB infections and 17 TB deaths before diagnosis. An additional 73 patients without active TB would receive inappropriate TB therapy. Implementing TB culture with solid media would avert 8 TB deaths (95% SI: 4, 15) and 17 secondary infections (95% SI: 6, 36) but would trigger inappropriate TB treatment of 44 additional patients (95% SI: 34, 57) with growth of NTM but not TB in culture. Replacing solid media with MGIT would avert one further death (95% SI: −1, 4) and five secondary infections (95% SI: -1, 12) but would generate 35 further inappropriate treatments (95% SI: 13, 59).

Compared with TB diagnosis using sputum smear alone, TB culture with solid media was estimated to avert 37 DALYs (95% SI: 13, 76) per 1,000 TB suspects, at a cost of $36 (95% SI: $25, $50) per TB suspect, or $962 (95% SI: $469, $2642) per DALY averted (Table 3). Replacing solid media with MGIT was estimated to avert an additional 8 DALYs (95% SI: −4, 23) per 1,000 TB suspects at a cost of $22 (95% SI: $8, $37) per suspect, giving an expected incremental cost-effectiveness ratio of $2751/DALY (95% SI: $680, dominated) compared to solid media. Increasing laboratory throughput threefold reduced the incremental cost, and thus also improved the incremental cost-effectiveness, of TB culture by more than 50% ($15 vs. $36 per TB suspect; $414 vs. $962 per DALY averted for solid media).

**Table 3:** Cost-Effectiveness of Expanded TB Culture per 1,000 TB Suspects.

Scenario	Cost (×1,000)^a^	DALYs Averted	Incremental Cost (×1,000)^a^	Incremental DALYs Averted	Incremental Cost-Effectiveness Ratio
*Throughput: 8 Patients/Week*					
Baseline	$130 ($94, $173)	0 (ref)	$0 (ref)	0 (ref)	0 (ref)
TB Culture: Solid Media	$166 ($129, $209)	37 (13, 76)	$36 ($25, $50)	37 (13, 76)	$962/DALY ($469, $2642)
TB Culture: MGIT	$188 ($147, $235)	45 (16, 91)	$22 ($8, $37)	8 (−4, 23)	$2751/DALY ($680, dominated^b^)
*Throughput: 24 Patients/Week*					
Baseline	$130 ($94, $173)	0 (ref)	$0 (ref)	0 (ref)	0 (ref)
TB Culture: Solid Media	$145 ($110, $189)	37 (13, 77)	$15 ($10, $22)	37 (13, 77)	$414/DALY ($198, $1141)
TB Culture: MGIT	$161 ($123, $206)	45 (16, 94)	$15 ($9, $22)	8 (−4, 23)	$1936/DALY ($600, dominated^b^)

DALY, disability-adjusted life year; TB, tuberculosis; MGIT, Mycobacteria Growth Indicator Tube.

a Costs are reported in 2006 US$ and include all costs to the national tuberculosis program other than the baseline evaluation.

b Higher cost and lower effectiveness than the alternative, thus precluding calculation of a meaningful cost-effectiveness ratio.

### Sensitivity Analysis

**Figure 2:**
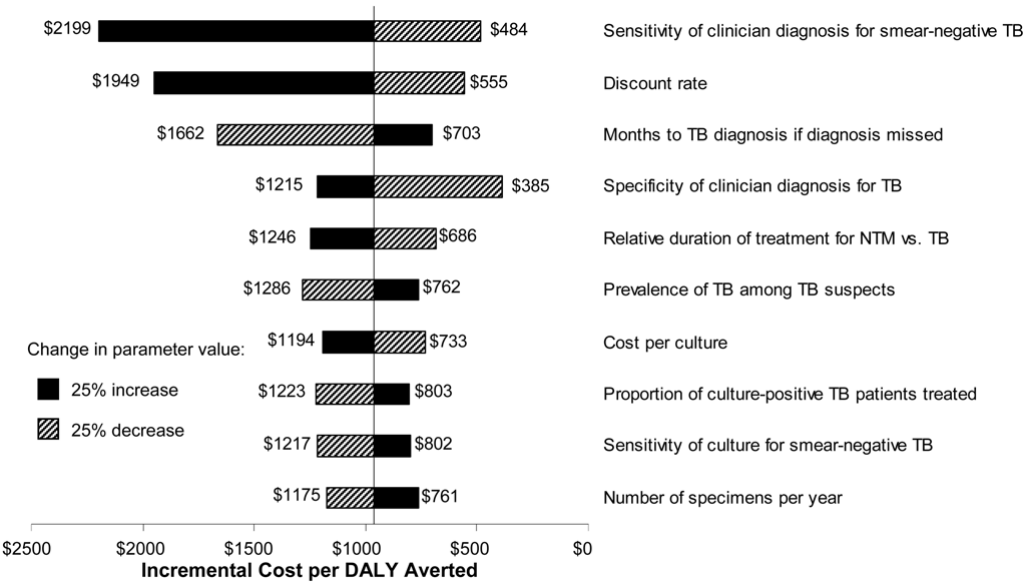
Results from One-Way Sensitivity Analysis. The effect of parameter variation on the estimated incremental cost-effectiveness ratio (ICER) for tuberculosis (TB) culture on solid media is shown. Results are similar for MGIT (data not shown). All parameters are varied by ±25% of their initial value (Table 2) except for the discount rate, which is varied from 0% to 7%. Only those parameters for which such variation changed the estimated ICER by ±20% are shown. Costs are in 2006 US dollars.

Results of one-way sensitivity analysis (Figure 2) show that the cost-effectiveness of expanded TB culture was more sensitive to the characteristics of the existing TB diagnostic system than the characteristics of culture itself. Decreasing the sensitivity of clinician diagnosis for smear-negative TB in the absence of culture from 70% to 42% improved the estimated incremental cost-effectiveness of TB culture from $2199 to $484 per DALY averted. Other conditions associated with a more favorable cost-effectiveness ratio included a low discount rate, longer delay to re-presentation by patients with undiagnosed active TB, high prevalence of smear-negative TB among TB suspects, and high rate of treatment based on positive cultures (Figure 2). When the three variables to which the model was most sensitive were set to values least favorable to TB culture, the incremental cost-effectiveness of culture (solid media) was $12,146 per DALY averted. The corresponding “best-case” cost-effectiveness ratio was $225 per DALY averted.

## Discussion

This operational evaluation of a centralized TB culture program serving HIV-positive patients in urban Brazil suggests that TB culture has substantial impact and reasonable cost-effectiveness when deployed in this setting. In this setting, we estimated that TB culture could avert an estimated 37 DALYs per 1,000 TB suspects and prevent 49% of all TB deaths occurring after initial presentation, at a cost of $962 per DALY averted (solid media). Upgrading from solid to liquid media averted an estimated 21% more DALYs at a cost of $2751/DALY. Rather than culture costs per se, the primary drivers of cost-effectiveness included clinical practices (e.g., empiric TB treatment), communications or patient follow-up (e.g., translating culture results into treatment), and TB prevalence.

Our findings suggest that TB culture can be implemented with reasonable cost-effectiveness in urban, middle-income Latin America. Although there is no universally-accepted threshold for considering an intervention “cost-effective,” the Commission for Macroeconomics and Health [21] has proposed that interventions whose cost per DALY averted is less than gross domestic product (GDP) per capita be defined as “very cost-effective.” By this criterion, TB culture among HIV-positive adults in Brazil (2007 GDP per capita: $9700 [22]) is very cost-effective. Although few studies have evaluated the cost-effectiveness of TB diagnosis in Latin America, other TB interventions have been shown to have a more favorable cost-effectiveness profile. For example, treatment of multidrug-resistant (MDR) TB in Peru was estimated to cost US$248 (converted to 2006 currency) per DALY averted [23]. Increasing throughput reduces the cost of TB culture substantially (Table 3), but similar or greater gains in cost-effectiveness could be achieved by targeting TB culture to regions with high TB prevalence and poor existing diagnostic sensitivity for smear-negative TB (Figure 2).

The overall impact of TB culture may be substantially greater than estimated here, as TB culture offers many clinical benefits beyond strict TB diagnosis. For example, we found that TB culture may triple the yield of bacteriologically-confirmed TB and avert 23% of TB transmission events occurring after the initial clinic visit. Furthermore, culture, unlike AFB smear, can discriminate NTM from TB and can identify drug resistant TB, thereby facilitating identification of optimal therapeutic strategies in individual patients. Finally, the number of TB suspects screened on a per-clinic basis in this study was relatively low, partially owing to gradual implementation and uptake; as clinics and clinicians adjust their practices to incorporate TB culture, both volume and cost-effectiveness are likely to improve.

Our results underscore the importance of translating culture results into clinical practice. In this study, despite weekly hand-delivery of all results to clinics, six of 17 patients (35%) with smear-negative TB were not initiated on TB therapy within 180 days of the clinic receiving a positive culture result. Four of these six patients had no entries in their medical records during that time, suggesting that failure to notify individual physicians and/or patients of positive culture results, or failure/inability of patients to report back to clinic, was responsible for the majority of such missed treatment opportunities. These results demonstrate that TB culture must be accompanied by effective post-laboratory procedures including information transfer, clinician education and training, and patient follow-up.

The goal of this analysis was to evaluate the impact and cost-effectiveness of TB culture as a whole; our small sample size precludes an authoritative comparison of MGIT versus solid media. Nevertheless, our results suggest that, if inoculated in parallel from a single clinical specimen, culture in solid media can achieve similar sensitivity to liquid media. In this analysis, five Lowenstein-Jensen slants achieved equivalent sensitivity to one MGIT tube. The additional benefit of MGIT over solid media resulted from MGIT's speed of diagnosis and the practice of re-processing MGIT cultures if contaminated. Of the 8 incremental DALYs averted by MGIT over solid media (Table 3), 4 were attributable to faster time to diagnosis, and 4 to increased sensitivity from re-processing of patient specimens.

TB culture resulted here in an unexpectedly high number of specimens positive for non-tuberculous mycobacteria, especially using MGIT. In fact, over 33% of positive cultures in TB suspects with negative sputum smears were identified as NTM rather than *M. tuberculosis*. Other studies have found increased NTM prevalence in a variety of developed-country settings [24]–[26], but the relevance of these findings to developing countries is unclear, as the relative prevalence of *M. tuberculosis* is higher. The present study highlights the importance of performing mycobacterial species identification in a developing-country setting and suggests that rapid methods to distinguish TB from other infections at the time of a positive culture result [27] may greatly enhance appropriate clinical decision-making.

This study has a number of limitations. First, our sample size of confirmed TB cases was small, and thus many of our parameter estimates are imprecise. However, our sensitivity analysis (Figure 2) suggests that the cost-effectiveness of TB culture is relatively insensitive to TB culture's accuracy or price; solid-media culture with 50% sensitivity for smear-negative TB and a cost of $50 per culture would still have an incremental cost-effectiveness of $1880 per DALY averted. Second, while our setting–a laboratory with existing TB culture capacity serving HIV patients in urban Brazil–is likely relevant to many middle-income regions in Latin America, our findings may not generalize to those settings (e.g., sub-Saharan Africa) where the co-epidemics of TB and HIV are most devastating. Third, certain aspects of our diagnostic algorithm may not be standard practice in other settings, making cost-effectiveness estimates more difficult to translate to local conditions. For example, we performed five parallel solid-media cultures (two with inhibitors) for each specimen, reflecting standard local lab practices in Rio de Janeiro. We also refrigerated unprocessed specimens for up to one week before transport to the laboratory. Both parallel processing and refrigeration would be expected to impact the estimated sensitivity of TB culture; Figure 2 shows the expected effect on cost-effectiveness of a 25% variation in this parameter. Finally, data for construction of the full cost-effectiveness model were limited. Thus, we could not adopt a societal perspective for our cost-effectiveness estimates, making our results less comparable to those from other studies [28]. However, the cost of TB culture to other members of society (e.g., patients) is likely small, and as mentioned above, our cost-effectiveness estimates may be conservative due to exclusion of ancillary benefits of TB culture. Other relevant data limitations include treatment outcomes, appropriate disability weights in HIV-positive patients undergoing TB treatment, and the true TB status of patients empirically treated for TB without a positive culture result.

In conclusion, this study suggests that TB culture for HIV-positive patients in urban Brazil may have substantial impact and reasonable cost-effectiveness. Cost-effectiveness was more sensitive to characteristics of the existing clinical infrastructure than to the cost or accuracy of TB culture itself; to be effective, TB culture must be implemented with programs to ensure effective communication between lab, clinic, and patient. Non-tuberculous mycobacteria accounted for an unexpectedly large proportion (>33%) of all positive culture isolates in this moderate TB prevalence setting, further emphasizing the need for rapid species identification methods to complement culture. TB culture is a potentially effective and cost-effective tool for use among HIV-positive patients in resource-constrained settings, but integration with existing clinical systems and strengthening of post-analytical processes are required to maximize impact.
